# Impact of lifestyle and comorbidities on seropositive rheumatoid arthritis risk from Korean health insurance data

**DOI:** 10.1038/s41598-022-06194-8

**Published:** 2022-02-09

**Authors:** JunSoo Ro, Se Hee Kim, Hae-Rim Kim, Sang-Heon Lee, Hong Ki Min

**Affiliations:** 1 Department of Health Policy and Management, Seoul National University College of Medicine, Seoul, Republic of Korea; 2 Division of Rheumatology, Department of Internal Medicine, Konkuk University Medical Center, 120-1, Neungdong-ro, Gwangjin-gu, Seoul, Republic of Korea; 3 Division of Rheumatology, Department of Internal Medicine, Research Institute of Medical Science, Konkuk University School of Medicine, Seoul, Republic of Korea

**Keywords:** Health care, Rheumatology, Risk factors

## Abstract

Rheumatoid arthritis (RA) is a systemic inflammatory arthritis in which primary prevention is key. However, the impact of lifestyle and comorbidities on RA development is unknown. Data from the Korean National Health Insurance Service (NHIS)-national sample cohort from 2002 to 2016 were used. At baseline, demographic characteristics, socioeconomic status, type of residential area, lifestyle behaviours (including exercise), and comorbidities (including the Charlson Comorbidity Index, CCI) were included. Cox regression analysis and Kaplan–Meier curves were used to evaluate the impact of lifestyle and comorbidities on seropositive RA occurrence. A total of 517,053 participants were included in the analysis for seropositive RA occurrence. Mean follow up duration was 71.5 and 142.3 person-month for seropositive RA occurrence group and non-occurrence group, respectively. Seropositive RA was diagnosed in 1,948 participants (0.37%) during follow-up. Cox regression analysis revealed that being aged between 40 and 79, a higher CCI, and hyperlipidemia resulted in elevated hazard ratios (HRs) for seropositive RA, whereas male gender, city residence, moderate alcohol consumption, high regular exercise and a BMI between 23 and 34.9 kg/m^2^ resulted in lower HRs. Using Korean NHIS data, the present study demonstrates that high-intensity regular physical exercise and moderate alcohol consumption are negatively associated with seropositive RA occurrence, which are modifiable lifestyle habits that might aid the primary prevention of seropositive RA.

## Introduction

Rheumatoid arthritis (RA) is an autoimmune-mediated, systemic arthritis, which significantly reduces quality of life and ability to work in affected patients^1^. Inflammatory arthritis including RA is difficult to treat and cause significant direct and indirect socioeconomic burdens to sufferers for the rest of their lives^2^. The incidence of seropositive RA has been increased^3^, and the mortality of RA patients are higher than general population^4^. These support the importance of preventing RA occurrence.

Due to the difficulty in treating RA, primary prevention of RA is preferable. However, several preventative interventions have been attempted during the preclinical state for RA, all of which have failed^1^. Several modifiable environmental factors for RA have been identified: smoking is the most well-known factor that influences joint damage progression in RA^5^, while alcohol consumption is inversely associated with seropositive RA development^6^, although excessive alcohol intake increases the risk of psoriatic arthritis in females^7^. Regular physical activity reduced risk of RA occurrence^8^. The impact of other modifiable environmental factors and controllable comorbidities on RA development has not yet been elucidated.

All residents in Korea are covered by national insurance. Patients with seropositive RA are exempt from medical expenses because seropositive RA is designated as rare intractable diseases (RID) by the National Health Insurance Service (NHIS)^9^. As a result, the Korean National Insurance Service and Review Board strictly monitor the diagnosis of seropositive RA, which makes diagnoses of seropositive RA relatively accurate and reliable. The NHIS produces sample cohort data, which are collected and stratified according to age, gender, type of insurance, and regional distribution of whole population in South Korea; this includes about 3% of all collected insurance data^10^. These data, known as the NHIS-National Sample Cohort (NSC), include insurance- and disease-related data. And national health screening data offers comorbidities and lifestyle habits including smoking, alcohol consumption and daily exercise. Therefore, merging NHIS-NSC data with national health screening data are suitable for investigating the influence of lifestyle and underlying comorbidities on the occurrence of specific diseases.

The aim of the present study was to identify the impact of combined comorbidities, socioeconomic status and lifestyle habits on seropositive RA occurrence using Korean NHIS-NSC data.

## Materials and methods

### Data sources

In this study, NHIS-NSC (2002–2016) data were used. The Korean NHIS is a mandatory single medical insurer system, and the National Health Information Database was developed by the Korean NHIS. Demographic information including sex, age, income, medical use, screening data (diet, exercise, alcohol consumption, smoking, etc.) and weight was collected. The NHIS-NSC sampled approximately 1.1 million individuals from the Korean population (of approximately 40 million) in 2002 and continued to collect cohort data until 2016, which is representative data of whole population in South Korea^10,11^. This study was conducted in accordance with the Declaration of Helsinki (1964 Declaration of Helsinki and its later amendments). Written informed consent was waived by the institutional review board of Konkuk University Medical Center because of the characteristics of NHIS data. This study was approved by the Institutional Review Board of Konkuk University Medical Center (approval number: KUMC 2020–03-018).

### Data extraction

An operational definition was used to increase diagnostic accuracy. To focus the analysis on newly diagnosed patients, we excluded patients who were already diagnosed with seropositive RA and were under medical care in 2002 and 2003. To register in RID program of Korean NHIS, the patients should be diagnosed as seropositive RA by satisfying either the 1987 American College Rheumatology (ACR) revised classification criteria of RA or the 2010 ACR/European League Against Rheumatism classification criteria^12,13^, and positive for either rheumatoid factor (RF) or anti-citrullinated peptide antibody (ACPA)^14^. Seropositive RA was defined as follows: 1) newly diagnosed with the Korean Classification of Diseases (KCD) code M058 as the main or first sub-diagnosis code; 2) having M058 as the main or first sub-diagnosis more than twice; and 3) being prescribed disease-modifying antirheumatic drugs for at least 6 months. The case satisfying all three criteria was defined as seropositive RA. This prescription was found using the Anatomical Therapeutic Chemical Classification System, searching specifically for the following medications: methotrexate (L01BA01, L04AX03), leflunomide (L04AA13), sulfasalazine (A07EC01), hydroxychloroquine (P01BA02), tacrolimus (D11AH01, L04AD02), infliximab (L04AB02), etanercept (L04AB01), adalimumab (L04AB04), golimumab (L04AB06), rituximab (L01XC02), abatacept (L04AA24), tocilizumab (L04AC07), tofacitinib (L04AA29), or baricitinib (L04AA37)^15,16^. Age was categorised within 10 year age groups (over 30 years old). Annual income was divided into three categories: low (70–100th percentile), intermediate (40–70th percentile), and high (> 40th percentile). Smoking status was categorised as current smoker or non-smoker, and ex-smoker was classified as non-smoker. Alcohol consumption was subdivided into four categories: non-drinker, mild drinker (1–4 cups per week), moderate drinker (5–10 cups per week) and heavy drinker (over 10 cups per week). Intensity of weekly exercise was categorised as follows: non-regular exercise, mild-intensity exercise (exercise for more than 30 min per week with normal breath), moderate-intensity exercise (exercise for more than 30 min per week with a little more breath than usual) and high-intensity exercise (exercise for more than 30 min per week with much more breath than usual). Obesity was defined according to body mass index (BMI, kg/m^2^) and categorised as underweight (< 18.5), normal (18.5–22.9), pre-obese (23–24.9), obese class I (25–29.9), obese class II (30–34.9), and obese class III (≥ 35)^17^. The age, annual income, smoking, alcohol, intensity of exercise, and BMI were checked at the time of enrolment in present cohort. The Charlson Comorbidity Index (CCI) was calculated^18^, and comorbidities such as hypertension (KCD I10-I15), diabetes mellitus (E10-14) and hyperlipidaemia (KCD E78.0–E78) were identified. The CCI and other comorbidities were checked at the end of follow-up.

### Statistical analysis and data management

Categorical variables were compared using a chi-squared test, and data are presented as numbers and percentage. Kaplan–Meier analyses with log-ranked tests were used to compare the cumulative incidence rate of seropositive RA. Cox regression analyses was performed to calculate hazard ratios (HRs) of lifestyle habits and comorbidities on seropositive RA occurrence. A two-tailed *P-*value < 0.05 was considered statistically significant. All tests except the Kaplan–Meier analysis were performed using the SAS Enterprise version 7.3 (SAS Institute, Inc., Cary, NC, USA). The Kaplan–Meier analysis was performed using R version 3.1.0 (R Foundation for Statistical Computing, Vienna, Austria).

### Significance and innovations

- Identifying modifiable, pathogenic environmental factors could aid primary prevention of seropositive RA.
- High intensity regular exercise, city residence and moderate alcohol consumption showed negative association with seropositive RA occurence.
- Exercising regularly and reducing comorbidities may help to prevent seropositive RA.

## Results

### Baseline demographics of seropositive RA occurrence and non-occurrence groups

**Figure 1:**
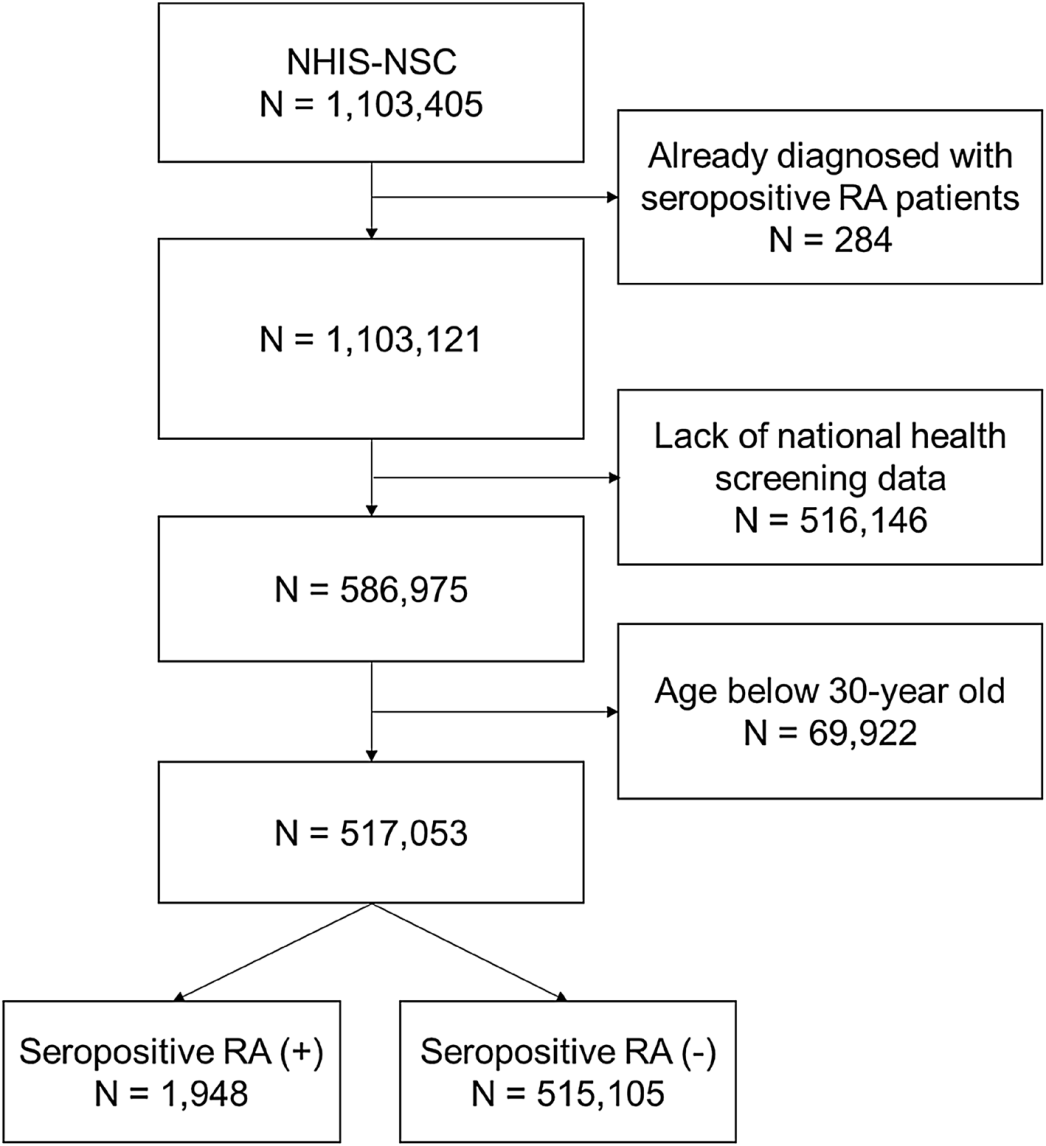
Flow chart for step-wise exclusion of participants from National Health Insurance Service-national sample cohort data.

At baseline, after excluding patients who were already diagnosed as seropositive RA, a total of 517,053 participants were included in seropositive RA analyses. Figure 1 demonstrate the flow chart for exclusion. During the follow-up duration, 1,948 (0.38%) participants were diagnosed as seropositive RA. The mean follow up duration for seropositive RA occurrence group and non-occurrence group were 71.5 and 142.3 person-month, respectively. The proportion of people who were over 50 years of age was higher in the seropositive RA occurrence group than in the non-occurrence group (79.6 vs. 59.4%, *P* < 0.0001). Other baseline characteristics of the seropositive RA occurrence and non-occurrence groups are summarised in Table 1.

**Table 1:** Baseline characteristics of seropositive rheumatoid arthritis (RA) occurrence versus non-occurrence groups.

	Seropositive RA occurrence group (n = 1,948)	Non-occurrence group (n = 515,105)	*P*
**Age category (years)**			< 0.0001
Age 30–39	76 (3.90%)	68,591 (13.32%)	
Age 40–49	321 (16.48%)	140,388 (27.25%)	
Age 50–59	589 (30.24%)	138,511 (26.89%)	
Age 60–69	507 (26.03%)	91,192 (17.70%)	
Age 70–79	358 (18.38%)	56,442 (10.96%)	
Age ≥ 80	97 (4.98%)	19,974 (3.88%)	
**Sex (female)**	1,446/1,891 (76.47%)	253,796/505,305 (50.23%)	< 0.0001
**Annual income category**			0.4895
70–100th percentile (Low)	871/1,947 (44.74%)	226,613/514,802 (44.02%)	
40–70th percentile	489/1,947 (25.12%)	143,408/514,802 (27.86%)	
> 40th percentile (High)	587/1,947 (30.15%)	144,781/514,802 (28.12%)	
**Residence**			0.0004
City	797/1,927 (41.36%)	230,941/509,215 (45.35%)	
Rural	1,130/1,927 (58.64%)	278,274/509,215 (54.65%)	
**Current smoker**	226 (11.60%)	125,422 (24.35%)	< 0.0001
**Alcohol consumption**			< 0.0001
Non-drinker	1,448/1,948 (74.33%)	285,620/515,104 (55.45%)	
Mild drinker	181/1,948 (9.29%)	62,200/515,104 (12.08%)	
Moderate drinker	102/1,948 (5.24%)	51,016/515,104 (9.90%)	
Heavy drinker	217/1,948 (11.14%)	116,268/515,104 (22.57%)	
**Daily exercise levels**			< 0.0001
Non-regular exercise	600 (30.80%)	131,919 (25.61%)	
Mild regular exercise	551 (28.29%)	132,200 (25.66%)	
Moderate regular exercise	276 (14.17%)	66,351 (12.88%)	
High regular exercise	521 (26.75%)	184,635 (35.84%)	
**BMI category (kg/m**^**2**^**)**			< 0.0001
Underweight (BMI < 18.5)	93 (4.77%)	17,742 (3.44%)	
Normal (BMI 18.5–22.9)	820 (42.09%)	195,586 (37.97%)	
Pre-obese (BMI 23–24.9)	426 (21.87%)	125,865 (24.43%)	
Obese class I (BMI 25–29.9)	541 (27.77%)	154,797 (30.05%)	
Obese class II (BMI 30–34.9)	58 (2.98%)	19,072 (3.70%)	
Obese class III (BMI ≥ 35)	10 (0.51%)	2,043 (0.40%)	
**Diabetes mellitus**	683 (35.06%)	120,706 (23.43%)	< 0.0001
**Hypertension**	1,000 (51.33%)	186,544 (36.21%)	< 0.0001
**Hyperlipidaemia**	1,045 (53.64%)	193,922 (37.65%)	< 0.0001

### Seropositive RA occurrence according to daily exercise and other associated risk factors

**Figure 2:**
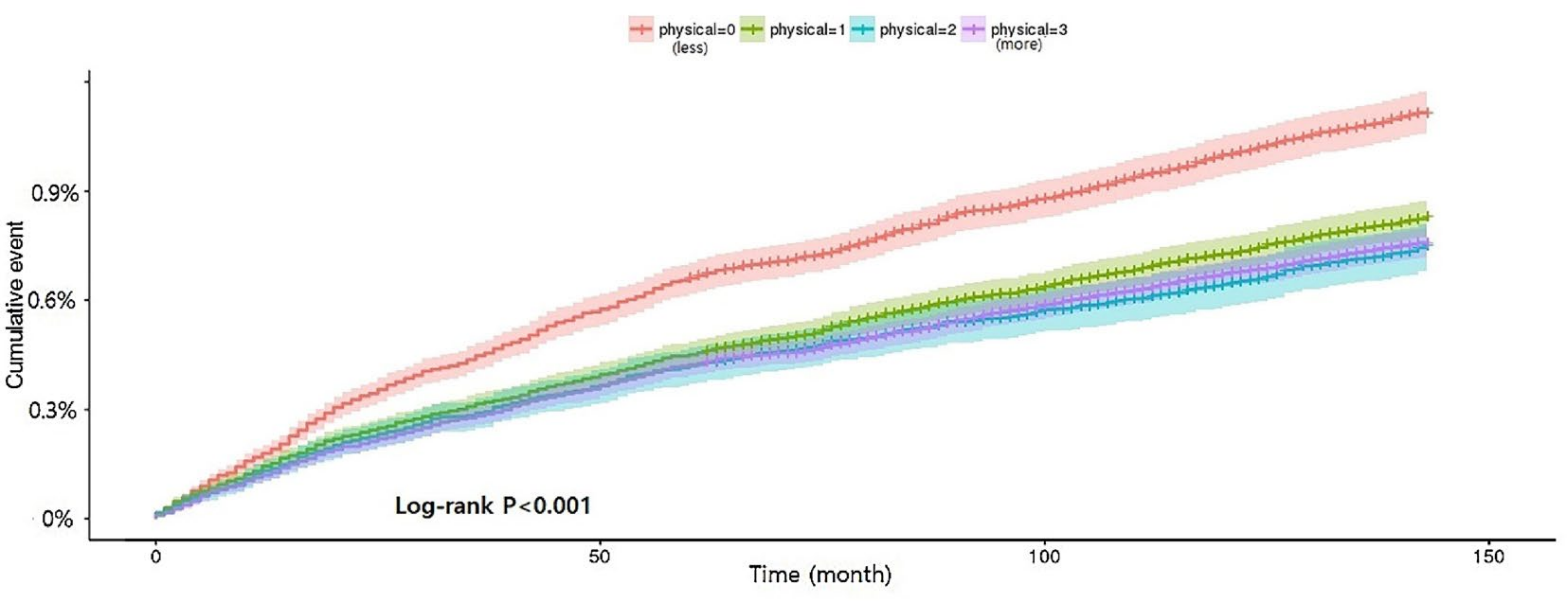
Cumulative incidence rate of seropositive rheumatoid arthritis according to daily physical exercise levels. Physical 0, non-regular exercise; physical 1, mild regular exercise; physical 2, moderate regular exercise; physical 3, high regular exercise.

The cumulative incidence of seropositive RA was analysed according to the intensity of regular exercise. Seropositive RA occurrence was significantly lower in the high-intensity regular exercise group than in the non-regular exercise group (*P* < 0.0001, Fig. 2). To measure other risk factors for seropositive RA occurrence, Cox regression analyses were performed. In multivariate Cox regression analyses of seropositive RA development, an age between 40 and 49 years (HR = 1.676), 50 and 59 years (HR = 2.445), 60 and 69 years (HR = 2.444), 70 and 79 years (HR = 2.063), a higher CCI (HR = 1.341) and having hyperlipidemia (HR = 1.226) resulted in significantly elevated HRs. Male gender (HR = 0.387), city residence (HR = 0.871), moderate alcohol consumption (HR = 0.810), high-intensity exercise levels (HR = 0.834), pre-obesity (HR = 0.780), obesity class I (HR = 0.774) and obesity class II (HR = 0.583) resulted in decreased HRs for seropositive RA occurrence (Table 2).

**Table 2:** Multivariate Cox proportional regression analysis for seropositive rheumatoid arthritis occurrence.

	HR	95% CI
**Age (years, N)**		
Age (30–39, N = 68,667)	(reference)	
Age (40–49, N = 140,709)	1.676	1.303–2.156
Age (50–59, N = 139,100)	2.445	1.909–3.131
Age (60–69, N = 91,699)	2.444	1.884–3.170
Age (70–79, N = 56,800)	2.063	1.563–2.772
Age (≥ 80, N = 20,071)	1.320	0.944–1.846
**Sex**		
Female (N = 255,242)	(reference)	
Male (N = 251,954)	0.387	0.340–0.441
**Annual income**		
70–100th percentile (Low, N = 227,484)	(reference)	
40–70th percentile (N = 143,897)	0.995	0.888–1.116
> 40th percentile (High, N = 145,368)	1.045	0.937–1.164
**Residence**		
Rural (N = 279,404)	(reference)	
City (N = 231,738)	0.871	0.794–0.956
**CCI (N = 517,053)**	1.341	1.327–1.354
**Smoking behaviour**		
Current non-smoker (N = 391,405)	(reference)	
Current smoker (N = 125,648)	0.934	0.789–1.106
**Alcohol consumption**		
Alcohol non-drinker (N = 287,068)	(reference)	
Alcohol mild drinker (1–4 cups per week, N = 62,381)	0.881	0.750–1.036
Alcohol moderate drinker (5–10 cups per week, N = 51,118)	0.810	0.657–0.999
Alcohol heavy drinker (over 10 cups per week, N = 116,485)	0.914	0.771–1.083
**Regular exercise levels**		
Non-regular exercise (N = 132,519)	(reference)	
Mild regular exercise (N = 132,751)	1.006	0.892–1.134
Moderate regular exercise (N = 66,627)	1.060	0.916–1.226
High regular exercise (N = 185,156)	0.834	0.737–0.944
**BMI category (kg/m**^**2**^**)**		
Underweight (BMI < 18.5, N = 17,835)	1.225	0.969–1.550
Normal (BMI 18.5–22.9, N = 196,406)	(reference)	
Pre-obese (BMI 23–24.9, N = 126,291)	0.780	0.692–0.880
Obese class I (BMI 25–29.9, N = 155,338)	0.774	0.690–0.869
Obese class II (BMI 30–34.9, N = 19,130)	0.583	0.440–0.772
Obese class III (BMI ≥ 35, N = 2,053)	0.837	0.431–1.625
**Diabetes mellitus (N = 517,053)**	0.959	0.858–1.071
**Hypertension (N = 517,053)**	1.108	0.988–1.242
**Hyperlipidemia (N = 517,053)**	1.226	1.104–1.362

## Discussion

The present study investigated the impact of lifestyle habits, comorbidities and socioeconomic status on the development of seropositive RA using Korean NHIS dataHigh-intensity exercise was negatively associated with seropositive RA occurrence. In addition, living in a city and moderate consumption of alcohol were negatively associated with seropositive RA occurrence. A higher CCI score was associated with a higher risk of seropositive RA development. These results reveal modifiable environmental factors that are associated with seropositive RA development for patients.

Little is known about the impact of lifestyle habits, including exercise, on the occurrence of seropositive RA. In the present study, we demonstrated that high-intensity exercise resulted in a decreased HR for seropositive RA. High-intensity of regular physical activity lowered the incidence of RA in two studies^8,19^. Although the method for dividing intensity of physical activity were differ from each study, however, the present study and aforementioned two studies identically showed that high-intensity regular exercise showed negative association with RA occurrence. Moderate alcohol intake (5–10 cups per week) was associated with a decreased risk of seropositive RA. In a meta-analysis, low to moderate alcohol consumption was associated with a lower risk of RA development than not drinking alcohol^20^, while another meta-analysis showed that alcohol consumption was negatively associated with ACPA-positive RA development but not ACPA-negative RA^6^. In prospective follow-up data of the Nurses’ Health Study II, excessive alcohol consumption (over 30 g of alcohol per day) increased the risk of psoriatic arthritis incidence, whereas low to moderate intake did not have a significant association^7^. These results cannot conclude the causality between lifestyle and occurrence of seropositive RA, and further basic research revealing the causality or mechanism of exercise and alcohol on seropositive RA pathogenesis should be performed.

Obesity is associated with an increased inflammatory burden^21^ and could therefore contribute to the development of inflammatory or autoimmune-mediated diseases^22,23^. However, Wesley et al. showed that obesity was negatively associated with ACPA-positive RA in males, whereas obesity increased ACPA-negative RA in females^24^. Another study demonstrated increased odds ratio for RA development in patients with obesity. Our results showed that the HRs of seropositive RA were lower in participants with pre-obesity and obesity class I and II. Conflicting results exist between present study and previous studies. These may arise from difference of ethnicity and cut-off value of BMI when defining obesity. . The present study and previous researchs^24,25^ suggest the heterogeneity of RA pathogenesis in which obesity may have different impact on RA development according to gender, ethnicity, and autoantibody status.

In a meta-analysis, smoking increased the relative risk (RR) of developing RA by 26% in the smoking group, which was higher in RF-positive cases (RR = 2.47) than RF-negative cases (RR = 1.58)^26^, suggesting that the impact of smoking may differ according to autoantibody status. Although our results suggest that smoking did not significantly change the HR for seropositive RA, this was an observational study. Therefore, to clarify the impact of smoking on the pathogenesis of RA, research using a larger sample size, which includes age, gender, autoantibody status and smoking status at baseline, should be conducted.

The CCI score was initially developed to predict 10 year mortality by collectively weighting various comorbidities by their seriousness^18^. Although a high prevalence of cardiovascular disease and pulmonary disease in patients with RA is already reported^27^, little is known about the impact of underlying comorbidities on the development of RA. The present study of insurance claim data demonstrated that a higher CCI score and underlying hyperlipidemia were associated with seropositive RA development. In addition, living in a city was associated with a lower HR for seropositive RA. The exact mechanism and influence of comorbidities or residence on RA pathogenesis is unknown, and the present study cannot show causality; however, this is the first study revealing a significant impact of comorbidities and residence on the occurrence of seropositive RA.

Several limitations exist in the present study. Most importantly, laboratory data for the autoantibodies RF and ACPA were lacking. Previously, the presence of RF or ACPA was associated with increased risk of RA development^28^. Unfortunately, the NHIS-NSC data do not offer these laboratory data which could reinforce the reliability of diagnosis, therefore, the validation of diagnosis were impossible in present study. NHIS-NSC is an ongoing project; thus data including autoantibodies may be available in the future. Second, NHIS-NSC data are observational, meaning conclusions relating to the causality of the factors on specific disease occurrence cannot be drawn. Therefore, basic research investigating the underlying mechanisms of specific factors on the pathogenesis of RA should be performed. Third, the NSC data represent a stratified sample taken from insurance claim data according to age, gender, type of insurance, and regional distribution of inhabitants; however, it still may not represent all insurance claim data. Fourth, we only included seropositive RA in the analyses; thus results cannot be generalised to seronegative RA. Fifth, Cox regression analysis depends on the duration of observation and prevalence of the diseases which could inflate the HRs. Therefore, the results of Cox regression analysis could not be applied to other ethnic population. Sixth, the validations of several variables such as smoking, alcohol consumption, intensity of exercise were impossible because these are recorded based on questionnaires. Also, these factors may change over follow up duration, however, the change of lifestyle, type of residential area were not recorded in follow up data. Finally, the amount / type of liquid of alcohol consumption, amount of smoking, and duration and intensity of exercise were not recorded in health screening survey, which strain the more detailed classification of alcohol consumption, smoking status, intensity of exercise.

In conclusion, we demonstrate that high-intensity regular exercise, moderate alcohol intake and residing in a city are negatively associated with seropositive RA occurrence. By contrast, combined comorbidities were positively associated with developing seropositive RA. These results suggest potential of managing comorbidities and modifying lifestyle habits may help to prevent the development of seropositive RA.
